# Not Parking Lots but Parks: A Joint Association of Parks and Transit Stations with Travel Behavior

**DOI:** 10.3390/ijerph16040547

**Published:** 2019-02-14

**Authors:** Keunhyun Park, Dong-Ah Choi, Guang Tian, Reid Ewing

**Affiliations:** 1 Department of Landscape Architecture and Environmental Planning, Utah State University, 4005 Old Main Hill, FAV 258, Logan, UT 84322-4005, USA; 2 College of Architecture + Planning, University of Utah, Salt Lake City, UT 84112, USA; dongah.choi@utah.edu (D.-A.C.); ewing@arch.utah.edu (R.E.); 3 Department of Planning and Urban Studies, University of New Orleans, New Orleans, LA 70148, USA; gtian@uno.edu

**Keywords:** mode choice, transit-oriented development, public space, first-mile and last-mile connection

## Abstract

Urban design literature says that public open space in a station area could promote walking and other types of physical activity, enhance place attractiveness, and increase property values. In the context of station areas, however, there is a lack of empirical studies on the relationship between the presence of parks and sustainable travel behavior, which is one of the primary goals of transit-oriented developments (TODs). This study examined the impact of park provision on transit users’ mode choice in three U.S. regions: Atlanta (GA), Boston (MA), and Portland (OR). This study utilized multilevel multinomial logistic regression to account for hierarchical data structures—trips nested within station areas—and multiple travel modes—automobiles, transit, and walking. After controlling for the built environment and trip attributes, this study showed that when there was a park, people were more likely to walk or take transit to access or egress a transit station. A transit station having a park nearby may provide a more pleasant first-mile/last-mile travel experience. This paper demonstrated that station areas need to incorporate more public space, an overlooked element in current TOD plans.

## 1. Introduction

Access to a park encourages physical activities including walking [1,2,3,4,5,6,7,8,9,10,11,12]. Furthermore, access to transit stations is associated with more transit use and walking, while it discourages automobile use [1,2,13,14,15,16,17,18]. As a result, a combination of transit stations with parks could have a synergistic effect on promoting sustainable travel behavior [19,20,21]. But there is a lack of empirical studies to test this theory. 

Transit-oriented developments (TODs) have gained popularity worldwide as a sustainable development strategy, in which public open space is an essential design element [19,21,22]. Calthorpe [19] described TOD as a mixed-use community within a walking distance of a transit stop and a core commercial area. Having been defined by various terms during the last two decades, the literature generally agrees on what constitutes a TOD: Development density, land use diversity, and walkability near transit modes that, when successfully implemented, turn out to maximize transit ridership and active modes of transportation and minimize automobile dependency [20]. Calthorpe [19] and certain of his successors [21,22,23] emphasized that TODs should contain open spaces that serve the needs of the surrounding community and provide a sense of community, safety, and comfort within a neighborhood. In a station area, parks and other types of public spaces are said to welcome transit riders, residents, and other visitors [19,21,22,23]. Public spaces have been referred to as “pearls on a string”, making transit access and egress trips more eventful [23]. They break a trip into shorter segments, as much as frequent intersections do. The literature to date, however, is still largely intuitive and anecdotal. 

**Figure 1:**
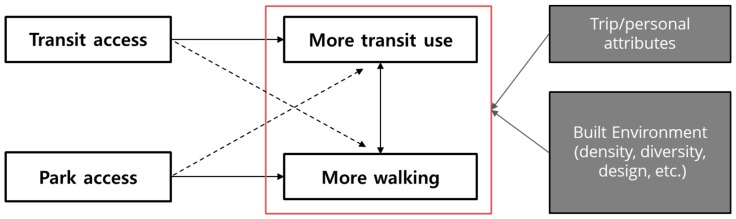
Conceptual framework of the relationship between a park near a station and travel behavior (solid line: Direct effect, dashed line: Indirect effect).

In this study, leveraging travel behavior data, we examined the relationship of park access near a transit station and travel mode choice. The travel behaviors examined were trips to and from transit stations. The statistical model used was multilevel multinomial logistic regression to account for hierarchical data structures—trips nested within station areas—and multiple travel modes—automobiles, transit, and walking. Figure 1 shows a conceptual framework of the current research. Transit access may lead to more transit use, and park access may encourage more walking, both of which correlate with each other. We hypothesized that a transit station having a park nearby may provide riders with a more pleasant environment and thus encourage walking to the station. The model included key built environment variables as controls, and also controls for trip and personal attributes.

Although public open space, like a park, is assumed as a multiplier boosting the benefits of TOD, the lack of empirical evidence makes TOD design guidelines abstract, which in turn fail to connect POS (public open space) theories with practices. The literature says that in general settings, access to public open spaces and specific attributes of POS, such as size and facilities, have a significant relationship with walking behavior [1,4,6,8]. In public health studies, the characteristics of accessible public open spaces could predict physical activity [3,5,6,7,8,24]. Nevertheless, transit officials have no solid baseline for public space that could make positive changes toward sustainable travel modes. 

Moreover, considering the vital role of first- and last-mile connections in promoting public transit use [25,26], the joint effects of parks and a transit station should be further understood. First- and last-mile refer to the distance covered before and after using a transit system. Converting station areas into walkable built environments has been an essential transportation mission across regions [27,28,29]. When municipalities aim at increasing transit ridership, lack of transit accessibility often discourages potential riders from reaching a transit station. A transit station having a park nearby may provide a more pleasant first-mile/last-mile travel experience, and thus, encourage transit riders to walk or take transit to access or egress a transit station. In this regard, the answer of how parks promote sustainable travel behavior in station areas could help local planners, developers, and decision makers establish criteria for park provision, which could be used when they (re)develop station areas or select the location of new transit stations or new parks.

## 2. Materials and Methods

### 2.1. Data

#### 2.1.1. Study Areas

Our study areas are three metropolitan regions in the U.S. that meet three criteria. First, they had to have household travel survey data with XY coordinates for trip origins and destinations. Second, a region had to provide land use databases at the parcel level so that we could study land use mix for the same years of the household travel surveys. Third, they had to have had a rail-based transit system before the survey was conducted. 

For the three regions chosen—Atlanta (GA), Boston (MA), and Portland (OR) (Table 1)— household travel surveys were done in 2011. Although they were conducted for different metropolitan planning organizations (MPOs), regional household travel surveys had a similar structure and questions, akin to those of the U.S. DOT’s National Household Travel Survey (NHTS). To gather comprehensive data on travel and transportation patterns, the survey data consistently included, but was not limited to, household demographic information, vehicle information, and data about one-way trips taken during a designated 24-hour period on a weekday, including travel time, mode of transportation, and purpose of trip information. The survey data had exact XY coordinates so we could geocode the precise locations of households and estimate the lengths of trips, whereas the NHTS provided geocodes of households only at the Census Tract level. The regional survey data were acquired from individual MPOs or state DOTs with confidentiality agreements.

Among the three regions, there were 374 rail-based transit stations according to the National TOD Database [30]. Transit types included heavy rail (87 stations), commuter rail (128 stations), and light rail (159 stations). 

Location data of local parks were gathered from governmental websites (e.g., metropolitan planning organizations, state GIS websites). To limit parks to those adjacent to the station, we used a threshold Euclidean distance of 200 feet (60 m). A total of 28 parks intersected with the 200-feet buffer and it was confirmed that they were built before 2011, the year of the surveys (Table 1). 

#### 2.1.2. Travel Behavior and Built Environment Data

From the household travel survey (HTS) data in the three regions, we extracted trips starting or ending within 100 feet (30 m) of a transit station. This study focused only on the first-mile/last-mile trips. There could be two cases: (1) A trip ending at a station *and* followed by a transit trip from the station (the first-mile connection) and (2) a trip starting at a transit station *and* following a previous transit trip (the last-mile connection). 

As a result, a total of 2209 trips (665 trips in Atlanta, 520 trips in Boston, and 1024 trips in Portland) were extracted. The outcome variable was a mode choice in each trip, which included three categories—walking, transit, and automobiles. Bikes and other modes were excluded because of their limited occurrence.

This study included “D” variables, which are built environment factors related to travel behavior [13]. Each D variable was measured for a ¼-mile buffer in network distance from a transit station. For the “density” variable, population and employment data at the Traffic Analysis Zone (TAZ) level were acquired from regional MPOs and summed to compute an overall activity density per square mile for each station area. 

For “diversity” variables, we computed an entropy index. Each region provided parcel maps so that we could calculate the proportion of the area of each land-use type, specifically residential, commercial, and public (e.g., public open space, governmental buildings, etc.) over the whole area of a ¼-mile buffer from each station. Then, the entropy index was calculated to measure the balance between three different land uses. The index ranged from 0, where all land was found in a single use, to 1 where land was evenly divided among the three uses [15].

For “design” variables, we computed intersection density as the number of intersections per square mile from street network shapefiles. For a “distance to transit” variable, transit stop shapefiles were acquired from each region and the number of bus and rail stops was divided by land area (square mile) for transit stop density. Transit type—light rail, commuter rail, and heavy rail—was also controlled. 

As trip attributes and personal attributes might affect a traveler’s mode choice, travel distance, trip purpose (work-related trip vs non-work trip), and the traveler’s age group (senior, child) were also modeled. The survey data had exact XY coordinates so we could geocode the precise locations of trip ends and measure the direct lengths of trips. Lastly, region dummy variables were also included. 

Descriptive statistics are presented in Table 2. Among the first-mile/last-mile trips, 69% was walking, 22% was transit trips, and 9% was automobile trips. The average trip distance was 1.62 miles. Work-related trips accounted for 30%. Among the travelers, 7% were seniors, and 4% were children. The number of stations included in the analysis was 138, not 374, because other stations did not have any first-mile/last-mile trips in the HTS data. Out of 138 stations, 27 stations (20%) had access to a park within 200 feet (60 m). Four built environment attributes—activity density, land use entropy, intersection density, and stop density—were measured for a ¼-mile network buffer around a station. A majority of the stations were served by light rail (54%), followed by heavy rail (36%) and commuter rail (11%). Stations in Portland (50%) were the most included in the analysis.

### 2.2. Statistical Method

**Figure 2:**
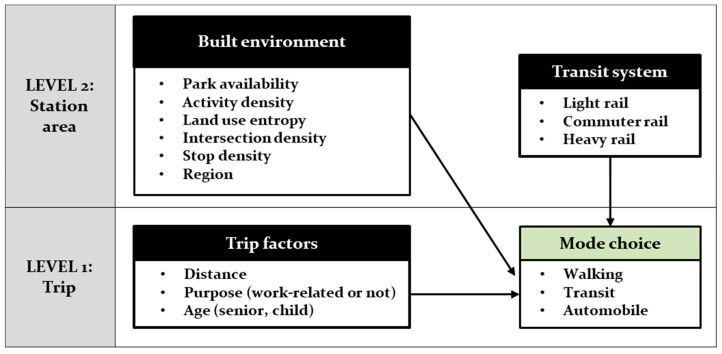
Multilevel model of mode choice in station areas.

The statistical approach of this study was a multilevel model (MLM) that fit the data structure. The data were hierarchical with individual trips nested within station areas (Figure 2). Trips in a station area shared built environment characteristics of the station area and this dependence violated the independence assumption of linear regression, in which residuals are independent of each other. Consequently, the standard errors of regression coefficients based on ordinary regression were underestimated. These limitations, however, were overcome by MLM, which accounts for dependence among observations and produces more accurate coefficient and standard error estimates [31]. The essence of MLM is to isolate the variance associated with each data level. In other words, MLM partitioned the variance between the trip level (Level 1) and the station area level (Level 2) and then sought to explain the variance at each level in terms of explanatory variables. 

The dependent variable, travel mode choice, had three categories: Walking, transit, and automobile. In MPOs or transportation agencies, travel mode choice is commonly modeled using a multinomial logit model [32,33]. As a discrete choice model structure, the multinomial logit model is used to predict travelers’ mode choices between two or more discrete alternatives—walking, transit, and automobile in this study. The choice is made depending on a traveler’s socioeconomic characteristics, the environmental condition where the trip occurs, or the trip characteristics themselves as variables [32,33]. We used a statistical software package, Stata 15.1 (StataCorp LP., College Station, TX, USA), with a *gllamm* (Generalized linear and latent mixed models) program to estimate our models. 

## 3. Results and Discussion

A multinomial logistic model can be interpreted similarly to binomial logistic regression because it is basically a series of comparisons between pairs of categories—in this case, walking versus automobile and transit versus automobile. The automobile mode was designated as the reference category. Table 3 provides coefficients—log-odds of a walking trip (or a transit trip) over an automobile trip for a one-unit change in the specific independent variable, standard errors, and p-values of the multinomial logistic regression model. 

When other factors were controlled, park provision had a positive association with the likelihood that a person would walk, not drive, to or from a transit station. The odds of a walking trip over an auto trip increased 3.55 times—the exponential of the corresponding regression coefficient, 1.268—with a park next to a station. Likewise, when there was a park next to a station, the likelihood that a person would take transit, not drive, increased. The odds of a transit trip over an auto trip increased 3.72 times—the exponential of 1.315—with a park provision. Both coefficients were significant at a *p* < 0.05 significance level.

To see if a larger park (or a larger area of parks) would have a stronger impact, we tested the total park area as a key independent variable instead of the binary park provision variable, but did not find a statistically significant association with either trip mode. 

At the trip level, the likelihood of a walking trip over an auto trip increased if it was work-related and decreased with trip distance. The opposite pattern was found in a comparison between transit and auto trips; the likelihood of a transit trip over an auto trip decreased in a work-related trip and increased with trip distance. Age group factors were not associated with mode choice in station areas. 

At station area level, activity density was positively associated with both walking and transit trips compared with auto trips. Transit stop density, the number of bus and rail stops per square mile, was positively associated only with the transit trip mode choice. Other built environment variables—land use diversity and intersection density—had no statistically significant relationship with mode choice. A trip was more likely to be by walking or transit for a light rail station than a commuter rail station. Regional differences were mostly not significant. 

The results conform to the literature that access to parks is associated with more walking [1,2,3,4,5,6,7,8,9,10,11,12]. However, this study differs from previous studies in that our finding was borne out in the particular context of station areas. It suggests that there is a value in constructing parks or public open spaces near rail stations for the purpose of promoting walking trips and, potentially, other physical activity. 

A more novel finding in this study was that a transit trip is also related to public space near a station. Instead of using park-and-ride, people tend to take a chain of transit trips (e.g., transferring from a bus to a rail transit, or vice versa) when they encounter a park during the first or last mile of a trip. Several well-known TODs have been designed with parks as “pearls on a string” leading through neighborhoods to transit stations, thus encouraging transit use. To our knowledge, this has been the first-of-its-kind empirical research supporting the practice. 

Lastly, the study provides practical implications for improving first- and last-mile connections. Although transit accessibility plays an essential role in transit ridership, strategic tools have focused on street elements such as highly visible traffic signs, marked or raised crosswalks, extended curbs, or new bike stations [25,26]. Based on the result of this study, the first- and last-mile strategy can extend further to include public space improvement near a transit station, and this environmental change may contribute to more sustainable travel behavior as well as more environment-friendly developments. 

This paper has some limitations and thus, calls for further study. First of all, there could be some important but missing variables affecting transportation mode choice. Omitted variables may include built environment characteristics around a station, such as land-use types, transit service quality, micro-level destination accessibility (e.g., public facilities, grocery stores, café, restaurants), and street safety, and other personal factors such as gender or race. Furthermore, urban design quality and parking availability, which are significant factors for walking and transit trips [13,34,35], were omitted because of a lack of available data.

In addition, not only park availability or park size, but also other attributes of a park, or different types of public space, might be important. The literature says that specific attributes of parks such as size, facilities, safety, natural features, or maintenance are associated with walking behavior [1,2,4,6,8,36,37,38,39]. These specific factors are particularly important in planning and designing a park or station area.

Second, this study only explored associations, not causality between park provision in TOD and mode choice. The main threat to causal inference is residential self-selection, meaning that some people may choose to live in station areas having parks or such amenities because they want to walk or take transit more. In this multi-region study, in which household travel surveys did not examine attitudes toward residential location, we could not control for self-selection effects. Nevertheless, most empirical studies of self-selection have found significant associations between the built environment and sustainable travel behavior, after controlling for self-selection influences [39,40,41,42].

When developing a station area, there could be a trade-off between more parks and other types of land uses such as high-density buildings. More or larger parks may mean less area for buildings and arguably, lower financial returns. This study showed that both park availability and development density are desirable goals. Further studies may need to explore this trade-off relationship. One approach could be using non-linear regression such as generalized additive models (GAM) [43]. A GAM model enables researchers to see where the optimal values of independent variables (e.g., park provision, activity density) maximizing transportation benefits are.

## 4. Conclusions

This study related park provision to travel mode choice near a transit station to see if a park promotes people walking or taking transit. The results confirmed the hypothesis: A park in a station area may work as a multiplier encouraging walking and transit trips while discouraging driving. 

By researching how parks promote sustainable travel behavior in station areas, this study has hopefully provided local planners, developers, and decision makers with substantial evidence on the benefits of incorporating parks into transit-oriented development projects. As suggested in the conceptual framework of this study (Figure 1), a transit station having a park nearby may provide riders with a more pleasant walking environment and thus encourage walking to the station.

Of course, the idea of TOD has always had as its goal more than enhanced transit ridership. With ample open space, TODs may increase physical activity, livability, a sense of community, property values, etc. When selecting the locations of new transit stations, availability of neighborhood parks can be among the criteria. Future studies validating the current research may build the evidence base for more successful and sustainable TOD planning and design.

## Figures and Tables

**Table 1:** Study regions and transit stations. ^1^

No	Region	Year of Survey	Heavy Rail	Commuter Rail	Light Rail	Total	Parks (200 ft; 60 m)
1	Atlanta, GA	2011	38	0	0	38	2
2	Boston, MA	2011	49	121	72	242	18
3	Portland, OR	2011	0	7	87	94	8
	Total		87	128	159	374	28

^1^ This table shows only transit stations that had opened before the survey year.

**Table 2:** Descriptive statistics.

Variable	Description	N	Mean	S.D.
Dependent variables				
Mode choice (categorical variable)	Walk	1524	-	-
	Transit	481	-	-
	Automobile	204	-	-
	Total	2209	-	-
Independent variables – trip/personal attributes				
Trip distance	Euclidean distance (miles) between trip origin and destination	2209	1.62	3.80
Work trip	Dummy variable of trip purpose (1 = work-related trip)	2209	0.30	0.46
Senior	Dummy variable of traveler age (1 = over 65 years old)	2209	0.07	0.25
Child	Dummy variable of traveler age (1 = below 15 years old)	2209	0.04	0.19
Independent variables – station area attributes				
Park provision	Dummy variable of park presence within 200 feet (60 m) from a station (1 = yes, 0 = no)	138	0.20	0.40
Activity density	Sum of population and employment per square mile in 1000s	138	46.89	62.13
Entropy	Land use entropy index ^1^	138	0.52	0.31
Intersection density	Number of intersections per square mile	138	272.22	150.03
Stop density	Number of transit stops (bus + rail) per square mile	138	262.63	302.13
LRT	Dummy variable of transit type (1 = light rail transit)	138	0.54	0.50
CRT	Dummy variable of transit type (1 = commuter rail transit)	138	0.11	0.31
HRT	Dummy variable of transit type (1 = heavy rail transit)	138	0.36	0.48
Atlanta	Dummy variable of region (1 = Atlanta)	138	0.26	0.44
Boston	Dummy variable of region (1 = Boston)	138	0.24	0.43
Portland	Dummy variable of region (1 = Portland)	138	0.50	0.50

^1^ The entropy index measured the balance between the three different land uses. The index ranged from 0, where all land was found in a single use, to 1 where land was evenly divided among the three uses. The entropy calculation was: Entropy = −[residential share ∗ ln(residential share) + commercial share ∗ ln(commercial share) + public share ∗ ln(public share)] / ln(3), where ln is the natural logarithm of the value in parentheses, and the shares are measured in terms of total parcel land areas [15].

**Table 3:** A multilevel multinomial logistic regression model of log-odds of a walking trip or a transit trip.

Independent Variables		Walk (vs. Automobile)			Transit (vs. Automobile)		
		Coefficient	Standard Error	*p*-Value	Coefficient	Standard Error	*p*-Value
Level 1 (trip)	Constant	3.540	0.974	<0.001	−0.719	1.048	0.492
	Trip distance	−1.533	0.101	<0.001	0.047	0.021	0.024
	Work trip	1.738	0.512	0.001	−2.680	0.724	<0.001
	Senior (>65)	−0.360	0.408	0.378	−0.373	0.428	0.384
	Child (<15)	0.171	0.522	0.743	0.435	0.506	0.390
Level 2 (station)	Park provision	1.268	0.616	0.040	1.315	0.617	0.033
	Activity density	0.001	0.001	0.026	0.001	0.001	0.015
	Entropy	0.482	0.591	0.415	0.880	0.601	0.144
	Intersection density	0.003	0.002	0.110	0.001	0.002	0.529
	Stop density	0.001	0.001	0.212	0.003	0.001	<0.001
	CRT (ref: LRT)	−2.504	0.787	0.001	−1.792	0.871	0.040
	HRT (ref: LRT)	−1.058	0.922	0.251	1.125	0.997	0.259
	Boston (ref: Atlanta)	−1.185	0.762	0.120	−0.538	0.752	0.475
	Portland (ref: Atlanta)	−1.613	0.985	0.101	−2.400	1.066	0.024

## References

[1] Handy S., Cao X., Mokhtarian P. (2005). Correlation or causality between the built environment and travel behavior? Evidence from Northern California. Transp. Res. Part D Transp. Environ..

[2] Pikora T., Giles-Corti B., Bull F., Jamrozik K., Donovan R. (2003). Developing a framework for assessment of the environmental determinants of walking and cycling. Soc. Sci. Med..

[3] Durand C.P., Andalib M., Dunton G.F., Wolch J., Pentz M.A. (2011). A Systematic Review of Built Environment Factors Related to Physical Activity and Obesity Risk: Implications for Smart Growth Urban Planning. Obes. Rev..

[4] Frank L., Kerr J., Chapman J., Sallis J. (2007). Urban form relationships with walk trip frequency and distance among youth. Am. J. Health Promot..

[5] Giles-Corti B., Broomhall M.H., Knuiman M., Collins C., Douglas K., Ng K., Donovan R.J. (2005). Increasing walking: How important is distance to, attractiveness, and size of public open space?. Am. J. Prev. Med..

[6] Humpel N., Marshall A.L., Leslie E., Bauman A., Owen N. (2004). Changes in neighborhood walking are related to changes in perceptions of environmental attributes. Ann. Behav. Med. Publ. Soc. Behav. Med..

[7] Neuvonen M., Sievänen T., Tönnes S., Koskela T. (2007). Access to green areas and the frequency of visits–A case study in Helsinki. Urban For. Urban Green..

[8] Owen N., Humpel N., Leslie E., Bauman A., Sallis J.F. (2004). Understanding environmental influences on walking: Review and research agenda. Am. J. Prev. Med..

[9] Park K. (2017). Psychological park accessibility: A systematic literature review of perceptual components affecting park use. Landsc. Res..

[10] Schipperijn J., Cerin E., Adams M.A., Reis R., Smith G., Cain K., Mitáš J. (2017). Access to parks and physical activity: An eight country comparison. Urban For. Urban Green..

[11] Siu V.W., Lambert W.E., Fu R., Hillier T.A., Bosworth M., Michael Y.L. (2012). Built environment and its influences on walking among older women: Use of standardized geographic units to define urban forms. J. Environ. Public Health.

[12] Toftager M., Ekholm O., Schipperijn J., Stigsdotter U., Bentsen P., Grønbæk M., Kamper-Jørgensen F. (2011). Distance to green space and physical activity: A Danish national representative survey. J. Phys. Act. Health.

[13] Ewing R., Cervero R. (2010). Travel and the Built Environment: A Meta-Analysis. J. Am. Plan. Assoc..

[14] Ding C., Wang D., Liu C., Zhang Y., Yang J. (2017). Exploring the influence of built environment on travel mode choice considering the mediating effects of car ownership and travel distance. Transp. Res. Part A Policy Pract..

[15] Ewing R., Tian G., Goates J.P., Zhang M., Greenwald M.J., Joyce A., Greene W. (2015). Varying influences of the built environment on household travel in 15 diverse regions of the United States. Urban Stud..

[16] Khan M., Kockelman K.M., Xiong X. (2014). Models for anticipating non-motorized travel choices, and the role of the built environment. Transp. Policy.

[17] Park K., Ewing R., Scheer B.C., Tian G. (2018). The impacts of built environment characteristics of rail station areas on household travel behavior. Cities.

[18] Park K., Ewing R., Scheer B.C., Khan S.S.A. Travel Behavior in TODs vs. Non-TODs: Using Cluster Analysis and Propensity Score Matching. Transp. Res. Rec. J. Transp. Res. Board.

[19] Calthorpe P. (1993). The next American Metropolis: Ecology, Community, and the American Dream.

[20] Reconnecting America (2008). Station Area Planning: How to Make Great Transit-Oriented Place (TOD 202).

[21] Ewing R., Bartholomew K. (2013). Pedestrian- and Transit-Oriented Design.

[22] Jacobson J., Forsyth A. (2008). Seven American TODs: Good practices for urban design in transit-oriented development projects. J. Transp. Land Use.

[23] Cervero R., Murphy S., Ferrell C., Goguts N., Tsai Y.-H., Arrington G.B., Witenstein N. (2004). Transit-Oriented Development in the United States: Experiences, Challenges, and Prospects (TCRP Report 102).

[24] Kaczynski A.T., Besenyi G.M., Stanis S.A.W., Koohsari M.J., Oestman K.B., Bergstrom R., Reis R.S. (2014). Are park proximity and park features related to park use and park-based physical activity among adults? Variations by multiple socio-demographic characteristics. Int. J. Behav. Nutr. Phys. Act..

[25] Boarnet M.G., Giuliano G., Hou Y., Shin E.J. (2017). First/last mile transit access as an equity planning issue. Transp. Res. Part A Policy Pract..

[26] Chandra S., Bari M.E., Devarasetty P.C., Vadali S. (2013). Accessibility evaluations of feeder transit services. Transp. Res. Part A Policy Pract..

[27] Los Angeles County Metropolitan Transportation Authority (2014). First/Last Mile Strategic Plan.

[28] Riverside Transit Authority (2017). First & Last Mile Mobility Plan.

[29] Utah Transit Authority (2015). First/Last Mile Strategies Study.

[30] National TOD Database. http://toddata.cnt.org/.

[31] Raudenbush S.W., Bryk A.S. (2002). Hierarchical Linear Models: Applications and Data Analysis Methods.

[32] Singleton P.A., Clifton K.J. (2013). Pedestrians in regional travel demand forecasting models: State-of-the-practice. Proceedings of the 92nd Annual Meeting of the Transportation Research Board.

[33] Clifton K.J., Singleton P.A., Muhs C.D., Schneider R.J. (2016). Representing pedestrian activity in travel demand models: Framework and application. J. Transp. Geogr..

[34] Willson R., Menotti V. (2007). Commuter parking versus transit-oriented development: Evaluation methodology. Transp. Res. Rec. J. Transp. Res. Board.

[35] Park K., Ewing R., Sabouri S., Larsen J. Street life and the built environment in an auto-oriented US region. Cities.

[36] McCormack G.R., Rock M., Toohey A.M., Hignell D. (2010). Characteristics of urban parks associated with park use and physical activity: A review of qualitative research. Health Place.

[37] Rioux L., Werner C.M., Mokounkolo R., Brown B.B. (2016). Walking in two French neighborhoods: A study of how park numbers and locations relate to everyday walking. J. Environ. Psychol..

[38] Zhai Y., Baran P.K. (2017). Urban park pathway design characteristics and senior walking behavior. Urban For. Urban Green..

[39] Cao X., Mokhtarian P.L., Handy S.L. (2009). Examining the impacts of residential self-selection on travel behaviour: A focus on empirical findings. Transp. Rev..

[40] Cao X.J., Xu Z., Fan Y. (2010). Exploring the connections among residential location, self-selection, and driving: Propensity score matching with multiple treatments. Transport. Res. Part A Policy Prac..

[41] Ewing R., Hamidi S., Grace J.B. (2016). Compact development and VMT—Environmental determinism, self-selection, or some of both?. Environ. Plan. B Plan. Des..

[42] Zhou B., Kockelman K. (2008). Self-selection in home choice: Use of treatment effects in evaluating relationship between built environment and travel behavior. Transp. Res. Rec. J. Transp. Res. Board.

[43] Hastie T.J., Tibshirani R.J. (1990). Generalized Additive Models.

